# Intranasal immunization with recombinant *Toxoplasma gondii* actin depolymerizing factor confers protective efficacy against toxoplasmosis in mice

**DOI:** 10.1186/s12865-016-0173-9

**Published:** 2016-10-06

**Authors:** Zhuanzhuan Liu, Litian Yin, Yaqing Li, Fei Yuan, Xiaofan Zhang, Jiazhi Ma, Hongmei Liu, Yanjuan Wang, Kuiyang Zheng, Jianping Cao

**Affiliations:** 1 Department of Pathogenic Biology and Immunology, Laboratory of Infection and Immunity, Xuzhou Medical University, Xuzhou, Jiangsu 221004 People’s Republic of China; 2 Department of Physiology, Shanxi Medical University, Taiyuan, Shanxi 030001 People’s Republic of China; 3 National Institute of Parasitic Diseases, Chinese Center for Disease Control and Prevention; Key Laboratory of Parasite and Vector Biology, MOH, China; National Center for International Research on Tropical Diseases, China; WHO Collaborating Center for Tropical Diseases, Shanghai, 200025 People’s Republic of China; 4 School of Public Health and Tropical Medicine, Southern Medical University, Guangzhou, Guangdong 510000 People’s Republic of China

**Keywords:** *Toxoplasma gondii*, Toxoplasmosis, Actin depolymerizing factor, Intranasal immunization

## Abstract

**Background:**

*Toxoplasma gondii* is an opportunistic protozoan closely associated with AIDS and vertical transmission. *T. gondii* actin depolymerizing factor (TgADF) plays an important role in actin cytoskeleton remodeling, and it is required to invade host cells. TgADF was a promising vaccine candidate. To observe the immunological changes and protective efficacy of recombinant TgADF protein (rTgADF) against *T. gondii* infection, we optimized the intranasal immunization dose of rTgADF and analyzed the survival rate and tachyzoite loads in mouse tissues after oral challenge with *T. gondii* tachyzoites.

**Results:**

rTgADF was prepared, purified, and combined with mouse anti-His antibody and rabbit anti-*T. gondii* serum. After intranasal immunization with 10 μg, 20 μg, 30 μg, or 40 μg of rTgADF, the 30-μg group elicited high levels of secretory IgA (sIgA) in nasal, intestinal, and vesical washes, raised IgG titres in the sera, strong proliferation of splenocytes, and increased secretion of IL-2 and IFN-γ when compared with the control group. When the mice were orally challenged with *T. gondii*, an increase in the survival rate (36.36 %) and a decrease in the tachyzoite loads in the liver (67.77 %) and brain (51.01 %) were observed.

**Conclusions:**

Our findings demonstrate that intranasal immunization with rTgADF can simultaneously trigger mucosal and systemic immune responses and protect the mice against *T. gondii* infection.

## Background

*Toxoplasma gondii* is an obligate intracellular protozoan that invades the nucleated cells of humans and many other mammals. *T. gondii* is the causative agent of toxoplasmosis. About one-third of world’s population has been infected by *T. gondii* [1]. Healthy individuals are usually asymptomatic; however, patients with AIDS or other immunosuppressive diseases may exhibit encephalitis and blindness or even die [2]. In pregnant women, toxoplasmosis can lead to abortion, stillbirth, fetal abnormalities, and congenital toxoplasmosis [3]. In addition, toxoplasmosis in domestic animals can result in substantial economic losses to the farming industry and threaten the health of humans who eat undercooked meat [4]. Currently, no specific medicine is available to prevent and cure the disease. Therefore, an effective vaccine against *T. gondii* infection is required [5].

The first commercial vaccine for toxoplasmosis was used to control abortion in sheep [6]. However, this vaccine from the live-attenuated S48 strain was not appropriate for humans because it could revert to the pathogenic strain [7]. Subsequently, many studies focused on crude inactivated antigen vaccines, subunit vaccines, and DNA vaccines, but there is no currently available vaccine to prevent *T. gondii* infection in humans [8, 9]. Further studies are required to screen for efficacious target antigens and deliver them to produce appropriate protective immunity via an optimal administration strategy [5].

*T. gondii* depends on gliding motility to invade host cells, and it needs power obtained from actin filament polymerization and turn-over [10]. *T. gondii* actin depolymerizing factor (TgADF) promotes efficient turn-over of actin filaments via weak severing of filaments and strong sequestering of monomers [11, 12]. As early as 1997, TgADF had been identified, and recombinant TgADF (rTgADF) could bind actin monomers and depolymerize F-actin [13]. BALB/c mice vaccinated with pVAX1-TgADF or rTgADF displayed specific humoral and cellular immune responses, prolonged survival time, and reduced brain cyst load [14, 15]. Therefore, TgADF is a promising vaccine candidate.

Previous studies have shown that the survival time of mice intramuscularly immunized with pVAX1-TgADF or rTgADF was longer than that of the control groups, but it was not statistically significant [14, 15]. In addition, the mice were challenged intraperitoneally with tachyzoites of *T. gondii*, which did not conform to the natural infection route. We needed to optimize the experimental conditions to improve the protective efficacy of the vaccine.

*T. gondii* infection is caused by the ingestion of an oocyst, cyst, or pseudocyst. The intestinal mucosal barrier provides the initial defense to prevent the infection. It is logical to pursue a vaccination approach that elicits a potent defense at the invasion site and maintains long-lasting protective immunity [16]. As a result, the nasal route is suitable for eliciting mucosal and systemic immune responses [17]. A previous study has shown that intranasal administration of CpG/TLA could provide a stable, pronounced, and effective vaccine against toxoplasmosis when compared with intramuscular administration [18]. In the present study, we prepared rTgADF, determined the optimal dose for intranasal immunization, and evaluated the protective efficacy against *T. gondii* infection in BALB/c mice.

## Methods

### Mice and *T. gondii* strain

Specific-pathogen-free female BALB/c mice (4–6 weeks of age) were purchased from the Chinese Academy of Medical Science Animal Center (Beijing, China). All mice were bred with food and water provided *ad libitum*. Tachyzoites of the *T. gondii* RH strain were stored in liquid nitrogen in the laboratory and maintained by intraperitoneal passaging in BALB/c mice, as described previously [19]. This study was conducted according to the Guidelines for the Laboratory Animal Use and Care Committee of the Ministry of Health, China and the Ethics Committee on Animal Research of Xuzhou Medical University (No. SCXK < SU > 2014–0003).

### Construction of the recombinant plasmid

Total RNA of *T. gondii* tachyzoites was extracted using the TRIzol reagent (life,CA, USA). The complete open reading frame (ORF) of TgADF (GenBank: U62146.1) was amplified using polymerase chain reaction (PCR) from the cDNA template with specific primers (forward primer: 5′-ACGCGGATCCATGGCGTCCGGAATGGGTG-3′ and reverse primer: 5′-ACCGCTCGAGCGCGAGGGGTGCGAGGTC-3′), in which *Bam*HI and *Xho*I were introduced (underlined). The following PCR conditions were used: 94 °C for 3 min, followed by 30 cycles of 94 °C for 30 s, 56 °C for 30 s, and 72 °C for 30 s and 72 °C for 7 min. The PCR products were cloned into the prokaryotic expression vector pET30a(+) to obtain pET-30a(+)-TgADF used to transform *Escherichia coli* DH5α competent cells. The positive plasmids were confirmed using restriction enzyme digestion, PCR, and DNA sequencing.

### Preparation of the recombinant protein

*E. coli* BL21/DE3 transformed with pET-30a(+)-TgADF were grown in Luria-Bertani (LB) medium supplemented kanamycin (50 μg/mL) at 37 °C until an optical density (OD_600_) of 0.4–0.6 was achieved. The expression was induced by adding 0.1 mM isopropyl-β-d-thiogalactopyranoside (IPTG) and maintaining the medium at 25 °C for 12 h. The bacterial pellets were harvested and lysed by sonication in an iced water bath for 15 min (power, 200 W; pulse on, 5 s; pulse off, 15 s). To prevent protein degradation, all subsequent steps were performed at 4 °C. After centrifugation at 8,000 × *g* for 15 min, the supernatant was mixed with Ni-NTA agarose (Qiagen, Germany) for 1 h. The column was washed with a buffer containing 20 mM imidazole, 50 mM NaH_2_PO_4_, and 300 mM NaCl. The target protein was eluted by increasing the imidazole concentration to 150 mM. The endotoxin was removed and detected using the ToxinEraser™ Endotoxin Removal Kit and Chromogenic End-point Endotoxin Assay Kit (Chinese Horseshoe Crab Reagent Manufactory, Xiamen, China). Purified rTgADF was stored at –80 °C until further use.

The expression and purification of rTgADF were analyzed using 12 % sodium dodecyl sulfate-polyacrylamide gel electrophoresis (SDS-PAGE), and rTgADF was transferred to polyvinylidene difluoride membranes, followed by blocking in 5 % skim milk for 1 h at 25 °C. After washing, the membrane was incubated with anti-His primary antibody (1:1000) or rabbit anti-*T. gondii* serum (1:200) at 4 °C overnight. Then, the membranes were probed with a horseradish peroxidase (HRP)-conjugated goat anti-rabbit IgG antibody (1:5000). The blot was visualized with enhanced chemiluminescence reagents.

### Intranasal immunization procedure and sample collection

The mice were randomly divided into 5 groups (8 mice per group). Four groups were intranasally administered with 10, 20, 30, or 40 μg of rTgADF that was separately dissolved in 20 μL of phosphate-buffered saline (PBS). The control group was immunized with PBS. On days 0, 14, and 21, the nostrils of the mice were slowly instilled with rTgADF protein solution (10 μL per nostril). Two weeks after the last immunization, blood was drawn from the retro-orbital plexus of the mice, centrifuged at 3,000 × *g* for 10 min, and the serum was isolated. The nasopharyngeal region, small intestine, and urinary bladder of all the mice were exposed and washed according to a previously described method [20]. Nasal, intestinal, and vesical washes were centrifuged at 3,000 × *g* for 10 min, and collected in Eppendorf tubes. All samples were stored at –20 °C for evaluation of antibody content.

### Determination of antibodies

Enzyme-linked immunosorbent assay (ELISA) was used to detect the levels of rTgADF-specific antibody, IgG in the serum, and secretory IgA (sIgA) in the mucosal washes [21]. A 96-well plate was coated with rTgADF at a concentration of 5 μg/mL. The serum was diluted 1:300 for IgG, and the mucosal washes were not diluted for sIgA. HRP-conjugated goat anti-mouse IgG (diluted 1:2500) or IgA (diluted 1:1000) was used as the secondary antibody. Absorbance was read at 492 nm by using a microplate reader (Bio-Tek, VT, USA). The average value of three independent experiments was recorded for each sample.

### Cytokine assays

The immunized mice were dissected under aseptic conditions. The spleens were harvested, gently ground, and then passed through stainless-steel meshes in PBS. Splenocyte suspensions were prepared after the removal of erythrocytes [22]. The cells were seeded in 24-well microtiter plates at a density of 1.5 × 10^6^ cells per well and stimulated with rTgADF (10 μg/mL). The plates were incubated in 5 % CO_2_ at 37 °C. Concentrations of IL-2 and IL-4 at 24 h, IL-10 at 72 h, and IFN-γ at 96 h in the harvested cell-free supernatants were assayed using ELISA kits (PeproTech, USA), according to the manufacturer’s instructions.

### Splenocyte proliferation assay

The isolated splenocytes were plated at 5 × 10^5^ cells per well in 96-well plates and incubated with a cell-culture medium, concanavalin A (Con A; 5 μg/mL), and rTgADF (10 μg/mL). Cell proliferative activity was measured using the cell counting kit CCK-8 (Dojindo, Japan), according to the manufacturer’s instructions. Absorbance was measured at 450 nm. The stimulation index (SI) was calculated as follows: SI = average OD_450_ values from stimulated cultures/average OD_450_ values from non-stimulated cultures.

### Parasite challenge

Two groups of BALB/c mice (30 mice per group) were immunized with PBS or 30 μg of rTgADF, as mentioned above. Twenty-two mice from each group were orally infected (intragastric administration) with a dose of 4 × 10^4^ tachyzoites for the acute assay. Thereafter, survival times of the infected mice were recorded daily. The other 8 mice per group were challenged with 1 × 10^4^ tachyzoites for the chronic model. Four weeks later, real-time PCR was used to quantify tachyzoite loads in the liver and brain for detecting the SAG1 gene, as previously described [23].

### Statistical analyses

SPSS 11.5 software was used for the statistical analysis. The results of antibody responses, lymphocyte proliferation, cytokine assays, and tachyzoite loads were presented as mean ± SD values for three independent experiments. The normality test was conducted using the Shapiro–Wilk test. Comparisons between groups were performed using one-way analysis of variance (ANOVA). The difference was considered statistically significant if *P* < 0.05. The survival rates of the mice were analyzed using Kaplan–Meier curves.

## Results

### Identification of rTgADF

**Fig. 1:**
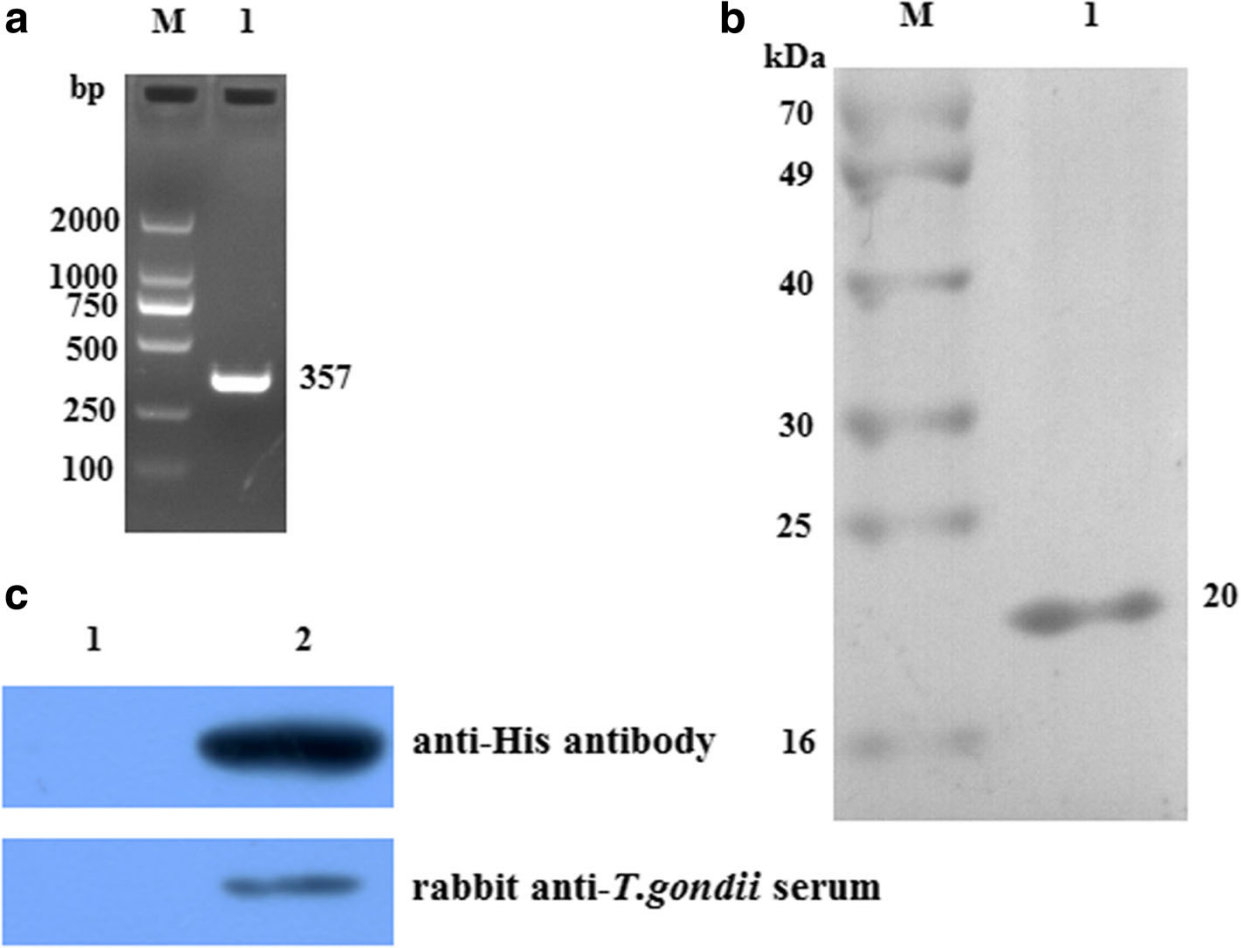
PCR detection of TgADF and SDS-PAGE and western blotting of rTgADF. **a** ORF of TgADF was amplified using PCR and examined with 1 % agarose gel electrophoresis. The length of the ORF was 357 bp. **b** TgADF was expressed in *E. coli*, purified using Ni-NTA agarose, detected with 12 % SDS-PAGE, and stained with Coomassie brilliant blue. Lane 1: Purified rTgADF displayed an apparent molecular mass of 20 kDa. **c** Western blotting showed that purified rTgADF (lane 2) reacted with anti-His antibody or rabbit anti-*T. gondii* serum, whereas *E. coli* containing the empty plasmid induced by IPTG did not show any band (lane 1)

The full-length ORF sequence of TgADF was amplified using RT-PCR, and the expected size was 357 bp (Fig. 1a). TgADF was inserted into pET-30a(+) and verified using PCR, restriction endonuclease digestion, and sequencing. After induction with 0.1 mM IPTG at 25 °C, rTgADF was expressed successfully in *E. coli* and then purified using Ni-NTA agarose. SDS-PAGE showed that the isolated protein was soluble and approximately 20 kDa, which is the molecular weight of the expressed rTgADF protein and a polyhistidine tag (Fig. 1b). Western blotting indicated that the purified rTgADF protein was able to combine with the anti-His antibody and rabbit anti-*T. gondii* serum (Fig. 1c).

### Evaluation of mucosal immune response

**Fig. 2:**
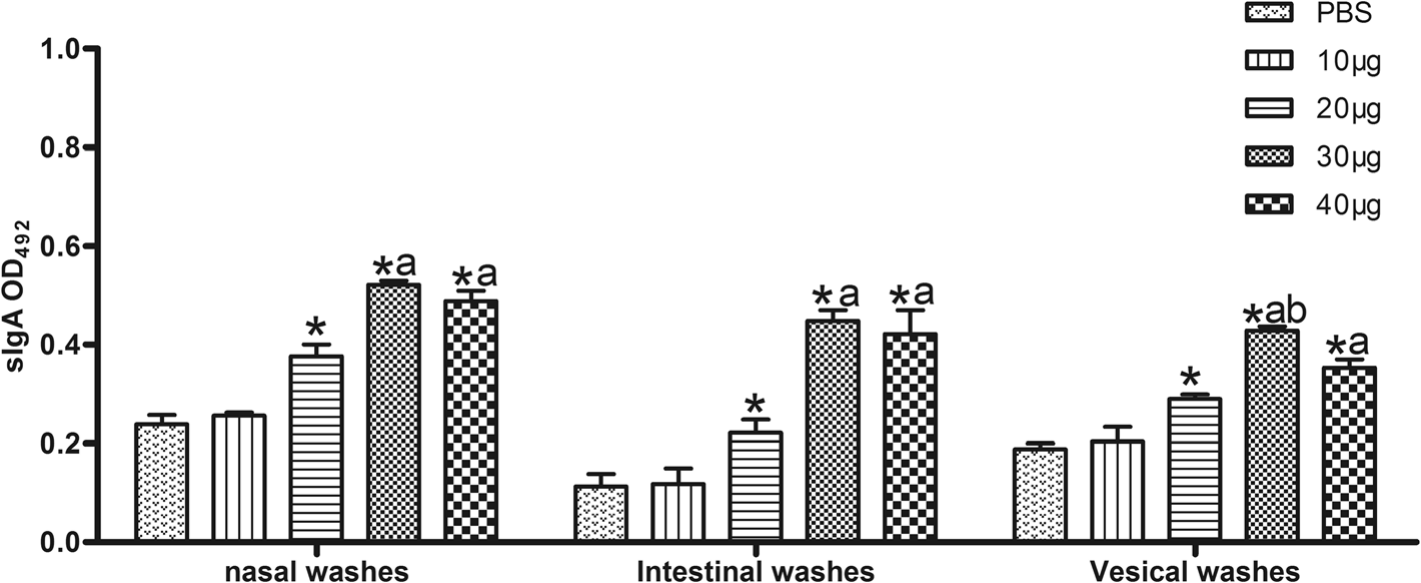
rTgADF-specific sIgA antibody responses in the mucosal washes. Nasal, intestinal, and vesical washes were harvested from the immunized (different doses of rTgADF) and control mice (PBS) two weeks after the last immunization. The sIgA antibodies were detected using ELISA. Results are expressed as mean ± SD (*n* = 8) for three independent experiments. *, *P* < 0.05 compared to the PBS group; a, *P* < 0.05 compared to the 20-μg group. b, *P* < 0.05 compared to the 40-μg group

We estimated nasal, intestinal, and vesical sIgA as indexes for mucosal immune response. Significantly higher levels of sIgA titers were observed in the nasal, intestinal, and vesical washes in mice immunized with 20, 30, or 40 μg of rTgADF than in the control groups (*P* < 0.05; Fig. 2). The sIgA levels in three mucosal washes in the 30- and 40-μg groups were prominently higher than that in the 20-μg group. Moreover, an apparent predominance of the 30-μg group over the 40-μg group was observed on the basis of the vesical washes. Results of the sIgA antibody levels in the mucosal washes demonstrated that 30 μg of rTgADF elicited the strongest response.

### Systemic immune response analysis

**Fig. 3:**
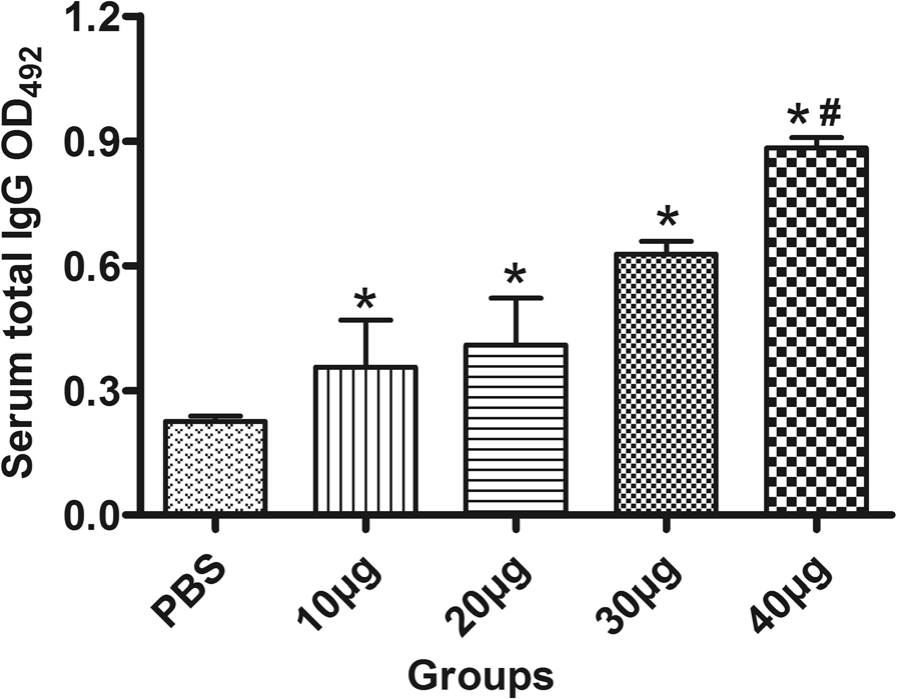
rTgADF-specific IgG antibody responses in the serum. Determination of IgG antibodies in serum samples collected from the immunized (different doses of rTgADF) and control mice (PBS) two weeks after the last immunization. Titers were the average of duplicate readings. Results are mean ± SD values (*n* = 8) for three independent experiments; *, *P* < 0.05 compared to the PBS group; #, *P* < 0.01 compared to the other immunized groups

**Fig. 4:**
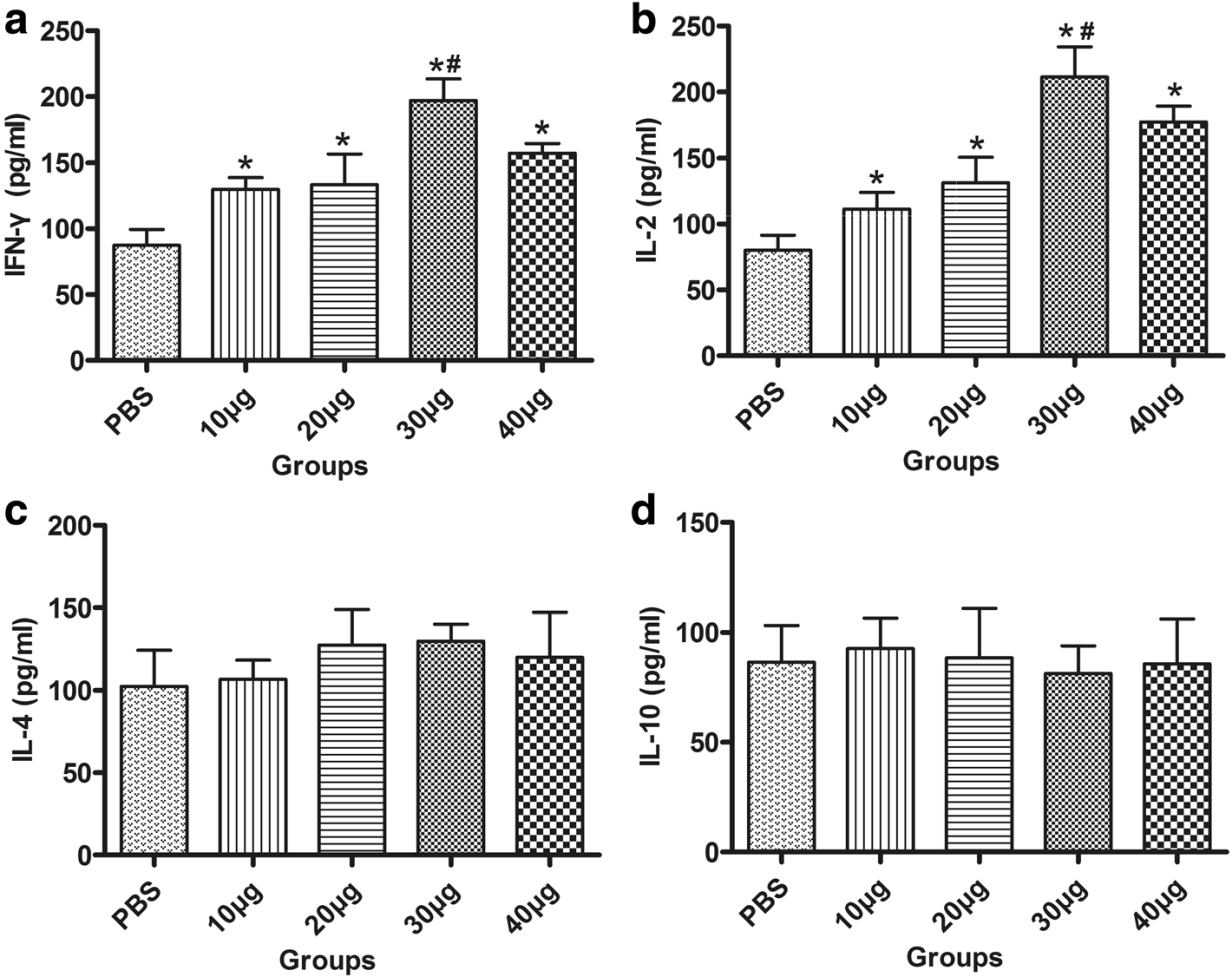
Cytokine concentrations of splenocytes from the immunized mice after stimulation with rTgADF. Splenocytes were collected from the mice two weeks after the last immunization and stimulated with 10 μg/mL of rTgADF. ELISA was used to examine the levels of IFN-γ (**a**), IL-2 (**b**), IL-4 (**c**), and IL-10 (**d**). Results are presented as means ± SD (pg/mL) for three independent experiments. *, *P* < 0.05 compared to the PBS group; #, *P* < 0.01 compared to the other immunized groups

Humoral and cellular immune responses were evaluated by analyzing IgG antibody levels in the serum, splenocyte proliferation, and cytokine production. High IgG titers were detected in the serum samples of all immunized mice (*P* < 0.05; Fig. 3). The OD values for IgG were continuously increased following an increase in the immunization dosage, and the 40-μg group showed the highest titer among all the immunized groups (*P* < 0.01). Splenocytes from the 30-μg and 40-μg groups exhibited a significantly greater proliferative response to rTgADF than splenocytes from the control group. No statistically significant differences were detected in the 10-μg, 20-μg, and control groups. Meanwhile, splenocytes from all groups proliferated to comparable levels in response to ConA (Table 1). In addition, we observed that splenocytes from all the immunized groups secreted significantly high levels of IFN-γ and IL-2 when compared with the control group, and the highest levels were elicited by 30 μg of rTgADF (Fig. 4a and b). In contrast, IL-4 and IL-10 levels displayed no significant changes between the immunized and control groups (*P* > 0.05; Fig. 4c and d).

**Table 1:** Splenocyte proliferative responses in immunized mice stimulated with rTgADF or ConA

Groups^a^	Proliferation (Stimulation Index)^b^	
	rTgADF	ConA
PBS	0.883 ± 0.0135	2.192 ± 0.1491
10 μg rTgADF	0.910 ± 0.0318	2.073 ± 0.1182
20 μg rTgADF	0.952 ± 0.0741	2.231 ± 0.1215
30 μg rTgADF	2.542 ± 0.1213*	2.284 ± 0.1743
40 μg rTgADF	2.391 ± 0.1912*	2.212 ± 0.1811

*P < 0.05 compared to the PBS group

^a^Splenocytes from mice (*n* = 8 per group) intranasally immunized with PBS and different doses of rTgADF were harvested two weeks after the last immunization and stimulated with rTgADF or ConA

^b^The level of splenocyte proliferation is expressed as the stimulation index (SI). Results represent mean ± SD values of three independent experiments

### Assessment of protection against *T. gondii* infection

**Fig. 5:**
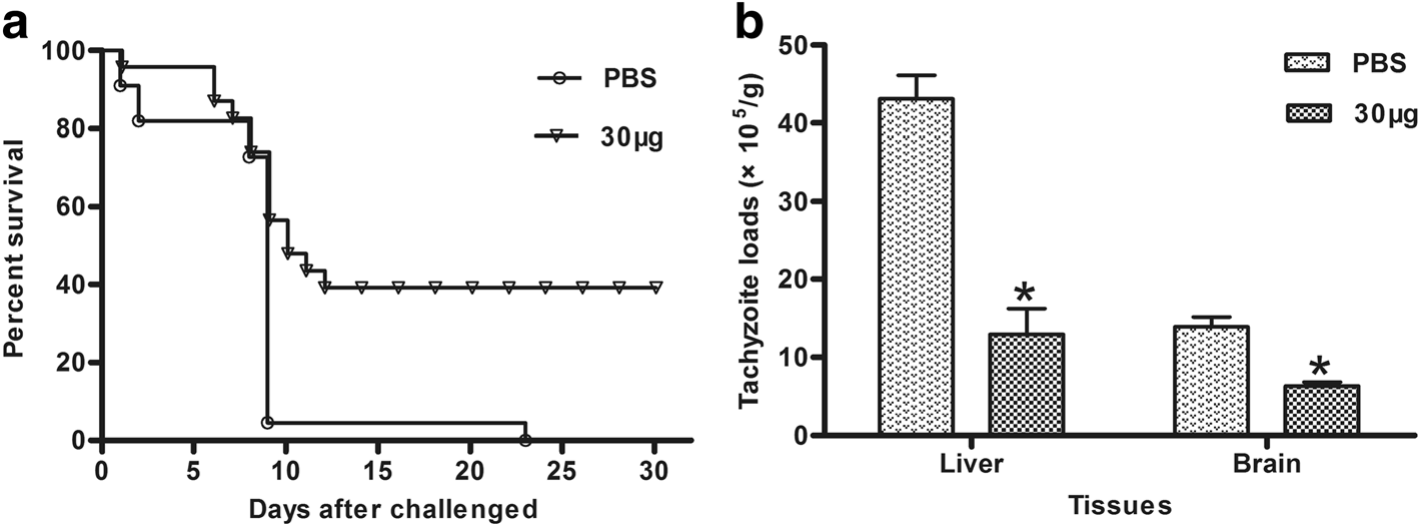
Protection of the mice immunized with rTgADF against oral challenge. The mice were intranasally immunized with PBS or 30 μg of rTgADF. **a** Survival curves of the mice (*n* = 22) challenged with 4 × 10^4^ tachyzoites (acute infection) and observed daily for mortality. **b** Abundance of tachyzoites was detected in the liver and brain of the mice (*n* = 8) after being challenged with 1 × 10^4^ tachyzoites (chronic infection). Results are expressed as mean ± SD values. *, *P* < 0.05 compared to the PBS group

Survival curves of the mice are shown in Fig. 5a. After the oral challenge with *T. gondii*, the mice showed symptoms such as vertical hair, lassitude, and lethargic and decreased appetite. Most of the mice in the control group were dead within 9 days post-infection, with the last mouse dying on the 23rd day. However, 36.36 % of the mice immunized with 30 μg of rTgADF remained alive 30 days post-infection. Similarly, rTgADF immunization protected against chronic infection. The tachyzoite load in the livers and brains was significantly lower in the immunized mice {13.89 ± 1.27 (10^5^/g) and 6.33 ± 0.43 (10^5^/g), respectively)} than in the control mice {43.09 ± 3.03 (10^5^/g) and 12.92 ± 3.30 (10^5^/g), respectively}, which showed a reduction in the tachyzoite load by 67.77 % in the liver and 51.01 % in the brain when compared with the control group (Fig. 5b).

## Discussion

In the current study, we investigated the protective capacities of rTgADF as an intranasal vaccine. The nasal route is easily accessible, requires low doses of an antigen, triggers mucosal immune responses at local and distant mucosa sites, and elicits systemic immune response [24]. Intranasally administered rTgADF was able to penetrate the nasal epithelium, where it was processed by the antigen-presenting cells. This process leads to the activation of T and B cells that develop into IgA plasma cells [17, 25, 26]. Mature sIgAs are compounded in the regional lymph nodes, and then enters circulation via the thoracic duct. They can reach distant mucosal sites (e.g. intestines, respiratory tract, genital tract, and salivary glands) [16, 17]. Interestingly, our experiments showed that rTgADF-specific sIgA levels in the nasal, intestinal, and vesical washes were the highest in the 30-μg group. The specific sIgA antibodies play a key role as the first line of defense against *T. gondii* infection [27].

Previous studies have indicated that nasal delivery of an antigen could also stimulate systemic humoral and cellular immune responses [28, 29]. Serum IgG has an important supporting role in systemic humoral immune function, and it is required for protecting the host cells against tachyzoite invasion [30]. In this study, all groups immunized with different doses of rTgADF showed significantly higher serum IgG levels than the control group. Although the 40-μg group showed the highest IgG titers, the best immune dose was ascertained by a combination of all the indicators, including splenocyte proliferation and cytokine and sIgA titers.

We evaluated the cellular immune response in the mice immunized with rTgADF. Splenocyte proliferation showed a 3-fold increase in the 30-μg and 40-μg groups. Expressions of IFN-γ and IL-2 were obviously increased in all immunized groups, especially the 30-μg group. IFN-γ is the major mediator of resistance against *T. gondii* [31]; it can activate macrophages and NK cells to engulf the pathogen and directly stimulate CD8^+^ T cells to exert cytotoxic effects [31, 32]. IL-2 is secreted by activated CD4^+^ Th1 cells, which promote T-cell proliferation, stimulate IFN-γ production, and enhance cytotoxicity [33]. Recombinant IL-2 administration enhances survival against a lethal challenge with *T. gondii* [34]. However, differences in IL-4 and IL-10 levels were not obvious between the control and immunized groups. These results showed that rTgADF activated Th1-typemediated immunity, which could provide protection against *T. gondii* infection.

On the basis of the mucosal and systemic immune indictors, the best dose of rTgADF was confirmed to be 30 μg, which was lower than the reported intramuscular dose (100 μg) [14]. Further, the protective efficacy of intranasal immunization with 30 μg of rTgADF against *T. gondii* was investigated. When the mice were orally challenged with tachyzoites, the survival rate of the immunized group was increased by 36.36 %, which was superior to that of mice immunized intramuscularly with the TgADF gene or protein [14, 15]. Compared with rTgPDI, rTgACT, and rTgMDH reported by our group, the survival rate attributable to rTgADF was close to that of rTgPDI but obviously lower than that of rTgACT and rTgMDH. In this study, the tachyzoite loads in the tissues were also significantly reduced, which was consistent with the results of the above-mentioned three proteins.

## Conclusions

In this study, our results demonstrated that intranasal immunization with rTgADF leads to the activation of both mucosal and systemic immune responses, resulting in protection against oral challenge with the RH strain of *T. gondii*. The immune efficacy may well be improved by combining rTgADF with other effective antigens such rTgMDH or adaptive adjuvants.

## Abbreviations

Con A: Concanavalin A
ELISA: Enzyme-linked immunosorbent assay
HRP: Horseradish peroxidase
IPTG: Isopropyl-β-d-thiogalactopyranoside
ORF: Open reading frame
PCR: Polymerase chain reaction
rTgADF: Recombinant TgADF protein
SDS-PAGE: Sodium dodecyl sulfate-polyacrylamide gel electrophoresis
SI: Stimulation index
sIgA: Secretory IgA
TgADF: *T. gondii* actin depolymerizing factor
